# Genome-wide identification and expression analysis of the *CBF/DREB1* gene family in lettuce

**DOI:** 10.1038/s41598-020-62458-1

**Published:** 2020-03-31

**Authors:** Sunchung Park, Ainong Shi, Beiquan Mou

**Affiliations:** 1 U.S. Department of Agriculture, Agricultural Research Service, Salinas, CA 93905 USA; 2 Department of Horticulture, University of Arkansas, Fayetteville, AR 72701 USA

**Keywords:** Gene ontology, Phylogeny, Abiotic

## Abstract

The C-repeat binding factor (CBF)/dehydration-responsive element binding (DREB1) proteins play a prominent role in freezing tolerance and are highly conserved in higher plants. Here we performed a genome-wide search of the *CBF/DREB1* gene family in lettuce (*Lactuca sativa* L.) and identified 14 members of the family with one member gene containing a non-sense mutation within the AP2 DNA-binding domain. A comprehensive phylogenetic analysis of the *CBF/DREB1* family members in 20 plant species from the Asterid or Rosid clade provided evidence that tandem duplication played an important role in the expansion of the *CBF/DREB1* family. Expression analysis showed that twelve of the lettuce *CBF* genes were responsive to low temperature (4 °C), and that three and six of them could also be responsive to salt and heat stresses, respectively. Unlike *Arabidopsis thaliana* whose members of the *CBF/DREB1* family respond only to a particular stress, lettuce CBFs provide wider protection from combinations of abiotic stresses. A global transcriptome analysis revealed distinctive temporal expression patterns among the cold-regulated genes in lettuce plants exposed to low temperature. Genes induced throughout the cold treatment are enriched in functions associated with protection from UV and high-light intensity and the genes suppressed after 7 days of cold exposure are enriched in photosynthesis-associated functions. These results provide insight into the molecular evolutionary properties of the *CBF/DREB1* gene family in lettuce and a reference for genetic improvement of the lettuce response to cold acclimation.

## Introduction

Freezing stress is a primary environmental factor that limits productivity and growth of plants. Many plants in temperate regions show an increase in freezing tolerance after exposure to low, non-freezing temperatures, a phenomenon called cold acclimation. The C-repeat binding factors (CBFs)/dehydration-responsive element binding (DREB1) proteins have been identified as key transcription factors in cold acclimation. In *Arabidopsis thaliana* (hereafter referred to as Arabidopsis), *CBF1*, *CBF2*, and *CBF3*^1,2^—also known as *DREB1b*, *DREB1c* and *DREB1a*, respectively^3^—are rapidly induced when plants are exposed to low temperatures, and they subsequently induce about 130 genes, referred to as the CBF regulon^4–6^. Expression of the CBF regulon brings about an increase in freezing tolerance, which is supported by the findings that overexpression of *CBF1*, *CBF2*, or *CBF3* leads to an increase in freezing tolerance without pre-exposing the transgenic plants to low temperature^5,7,8^ and attenuation of the CBF pathway in plants exposed to low temperature results in a decrease in freezing tolerance^6,9^. The mechanisms by which the CBF regulon increases freezing tolerance are not fully understood, but involve the function of genes encoding cryoprotective polypeptides^10,11^ and enzymes associated with the synthesis of low molecular weight cryoprotectants such as proline and raffinose^12,13^.

Arabidopsis *CBF1*, *CBF2*, *and CBF3* are linked tandemly in chromosome 4 and are very similar to each other in protein sequences with > 86% identity in full-length and >95% over the AP2-domain, suggesting that the *CBF* genes evolved through tandem duplication. In addition, the nonsynonymous (Ka) and synonymous (Ks) substitution ratio between them is significantly less than 1^14^. In general, a Ka/Ks ratio greater than one indicates positive selection, and the ratio close to 1 indicates neutral selection, while the ratio less than 1 indicates purifying selection that leads to limited functional divergence of the duplicated genes. Thus, the result implies that the CBFs are under the influence of purifying selection and have not diverged much in function after duplication. Consistently, CBF1, CBF2, and CBF3 regulate similar sets of genes as overexpression of *CBFs* induces similar sets of genes^6^ and the mutation studies using CRISPR genome-editing technology suggests their functional redundancy as the three Arabidopsis CBFs are required for a full capacity of freezing tolerance^15–17^.

The *CBF/DREB1* genes are well conserved in flowering plants^18^. Homologs of Arabidopsis *CBF* genes have been identified and characterized in several species including maize^19^, rice^20^, barley^21,22^, wheat^23^, tomato and rapeseed^18^, soybean^24^, and oat^25^. The CBF/DREB1 homologs contain an APETALA2/ethylene-responsive element binding factor (AP2/ERF) DNA-binding motif^26^. The CBF pathway has been identified as a locus contributing to freezing tolerance in Arabidopsis^27,28^, *Medicago*^29^, barley^30^, and wheat^31^. Thus identification of the CBF pathway genes would be a critical step towards improvement of freezing tolerance in agricultural crops.

The Arabidopsis genome encodes three other genes closely related to the *CBF* genes, *CBF4*, *DDF1*, and *DDF2*. Previously, Nakano *et al*.^32^ showed through a phylogenetic analysis that these six genes constituted a subfamily, designated IIIc, which is part of a larger monophyletic group III in the AP2/ERF superfamily in Arabidopsis. The members of IIIc subfamily (i.e. CBF/DREB1 subfamily) are separated from other AP2/ERF family members by the CBF signature sequence motifs (PKK/RPAGRxKFxETRHP and DSAWR) flanking the AP2 domain^18^. Despite high sequence similarity and similar sets of target genes between the family members^33^, they appeared to be involved in different abiotic stresses. For example, CBF1, CBF2, and CBF3 respond only to low temperature and are involved in cold acclimation; CBF4 is involved in drought tolerance^33^; and DDF1 and DDF2 are involved in high-salinity tolerance^34^. These results suggest that much of their functional divergence lies in different responsiveness to stress rather than protein sequences. Thus, the identification of the CBF pathway associated with freezing tolerance would require both phylogenetic analysis and expression analysis.

Lettuce is an important vegetable crop, with health benefits attributed to vitamin C, phenolic compounds, and fiber content^35,36^ and recently the genome sequence of a lettuce cultivar ‘Salinas’ has been released^37^. Lettuce is a cool season vegetable that can grow in regions with mild winters with an optimal temperature range of 13 °C to 18 °C^38^. However, brief exposure to frost during the winter season may reduce quality of lettuce. Frost damage often occurs in the winter lettuce production areas of Imperial Valley, California and Yuma, Arizona^39,40^. Freezing temperatures cause blisters and peeling of lettuce leaves, leading to decay and rot. Damaged leaves also provide entrance for plant pathogens. Improved freezing tolerance is an important long-term lettuce breeding goal for winter production. As a first step toward the goal, we identified the *CBF/DREB1* gene family in lettuce through a comprehensive phylogenetic analysis and characterized expression of the genes under various stress conditions to determine their associations with abiotic stress response. In addition, we investigated the cold-regulated (COR) genes that might play important roles in the process of cold acclimation in lettuce. These results provide insight into the mechanism of freezing tolerance as well as evolution of the *CBF/DREB1* genes in lettuce.

## Results

### Identification of *CBF/DREB1* gene family in lettuce

The *CBF/DREB1* genes in Arabidopsis belong to a subfamily, namely, the group IIIc with six gene members^32^. We used all six Arabidopsis genes as queries to search the protein database of lettuce (genome version 8) using the BLASTP method. To determine the *CBF/DREB1* gene family through a comparative phylogenetic analysis, we expanded the search to 19 other plant species. The 19 species were selected from the Asterid and Rosid clades that represent the two largest clades in flowering plants^41^: nine species including Arabidopsis were selected from the Rosid clade, and ten species were selected from the Asterids, which also includes lettuce. (Table S1).

Based on sequence similarity (E-value <1e-20) with the Arabidopsis proteins and the presence of an AP2 DNA-binding domain, we detected 652 *CBF*-like genes from the 19 species and lettuce (Table S2**;** see Materials and methods). The number of genes varied among species, ranging from 19 genes from chili pepper (*Capsicum annuum*) or sesame (*Sesamum indicum*) to 65 genes from cotton (*Gossypium hirsutum*). Additionally, 38 genes were identified from lettuce; 22 genes were identified from Arabidopsis that correspond to the previously identified genes of the Arabidopsis group III except a pseudo gene^32^. In general, the number of *CBF*-like genes in each species correlated with the total number of genes in its genome with an R2 of 0.75 (Table S3**)**.

**Figure 1:**
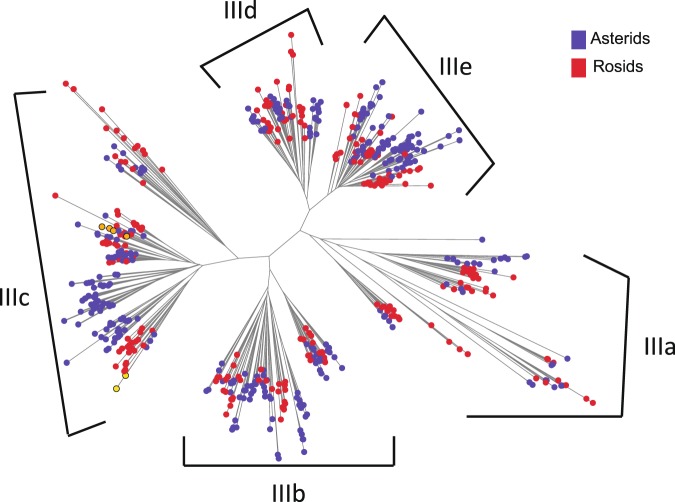
Phylogenetic analysis of the 652 *CBF*-like genes from 20 species including lettuce and Arabidopsis. The eleven species in the Asterid clade including lettuce are marked in blue circles; the nine species in the Rosid clade including Arabidopsis, in red circles; Arabidopsis CBF1–4, in gold circles; and Arabidopsis DDF1–2, in yellow circles. Five clusters, IIIa-e are named following the classification of the Arabidopsis AP2/ERF family by Nakano *et al*.^32^.

To determine the *CBF/DREB1* gene family in the *CBF*-like genes, we conducted a phylogenetic tree analysis with the amino acid sequences of the genes. The phylogenetic tree distinguished five clusters (IIIa, IIIb, IIIc, IIId, and llle) (Fig. 1), consistent with the previous classification in Arabidopsis^32^. All five clusters contained genes from both Asterid and Rosid species, indicating that the clusters diverged prior to evolutionary separation of the Asterid and Rosid clades. The cluster IIIc corresponding to the *CBF/DREB1* family had 198 genes in total including the six Arabidopsis *CBF/DREB1* genes (Table S2). There were 13 lettuce genes in the IIIc cluster, twelve of which had predicted protein sizes ranging from 197 to 231 amino acids, whereas one gene (*LsCBF7*; *Ls9g54981*) had a predicted protein size of 65 amino acids. The *LsCBF7* had a nonsense mutation within the AP2-DNA binding domain that resulted in a truncated protein (Fig. S1). To ensure that all the *CBF/DREB1* family members in lettuce genome were identified, we searched the lettuce genome sequence using the tBLASTN method with protein sequences of the identified 13 genes as queries. A new gene (*Ls9g54101.1*) was found with a significant E-value of 3E-98, which was annotated as a splicing variant of the representative *Ls9g54101.3* locus in the lettuce genome v8. The two splicing variants are, however, located in separate genomic regions (Fig. S2a) and encode similar but distinct proteins (Fig. S3), suggesting that they are different genes. Consistently, our RNA-seq analysis indicated independent transcriptions from the splicing variants as RNA-seq reads were mapped exclusively to each of the variants (Fig. S2b). Thus, we included the new gene as a member of the lettuce *CBF/DREB1* family. In addition, the *LsCBF8* gene sequence was incomplete in the genomic database, and we corrected it by Sanger sequencing **(**Fig. S4). Finally, a total 14 genes were identified and named consecutively from *LsCBF1* to *LsCBF14*, according to their chromosomal locations (Table 1).

**Table 1:** List of *CBF/DREB1* genes in lettuce.

Gene.Name	^a^Gene.Model	Protein.Length (amino acids)	^b^Location in Chromosome (Chr: start - end)
LsCBF1	Ls2g130641	197	2: 210322269–210322862
LsCBF2	Ls6g13081	209	6: 10672781–10673410
LsCBF3	Ls9g53921	231	9: 62232796–62233491
LsCBF4	Ls9g53960	223	9: 62282593–62283264
LsCBF5	Ls9g53101.3	222	9: 62497515–62498183
LsCBF6	Ls9g53101.1	222	9: 62505221–62505889
LsCBF7	Ls9g54581	69	9: 62769828–62770481
LsCBF8	Ls9g54900	220	9: 63083658–63084320
LsCBF9	Ls9g54880	220	9: 63083658–63084320
LsCBF10	Ls9g54860	220	9: 63099989–63100651
LsCBF11	Ls9g54780	210	9: 63158427–63159059
LsCBF12	Ls9g54680	222	9: 63227326–63227994
LsCBF13	Ls9g114680	210	9: 186836649–186837281
LsCBF14	Ls9g114720	214	9: 187031234–187031878

^a^Name of gene model was modified from the annotation of the lettuce genome v8, which consist of: (1) the prefix ‘Ls’ indicating the lettuce species abbreviated from L. sativa; (2) one digit number indicating its chromosome (1–9); (3) a letter, ‘g’ indicating that the name is for a gene; and (4) a 4–6 digit number from the lettuce genome v8 that is assigned uniquely to each gene.

^b^Locations represent the coding region of gene.

The 14 *LsCBF* genes showed 41–98% identity to each other at the amino acid level in full-length, and 71–100% identity for the AP2 DNA-binding domain (Fig. S5**)**. The CBF/DREB1 signature sequence motifs^18^ were also highly conserved with a few variations. An arginine (R) was present in place of the proline (P) at the fourth residue of the first motif (PKK/RPAGRxKFxETRHP), and in the second motif (DSAWR), a valine (V) was more common at the third position.

### Phylogenetic analysis of the *CBF/DREB1* gene family

**Figure 2:**
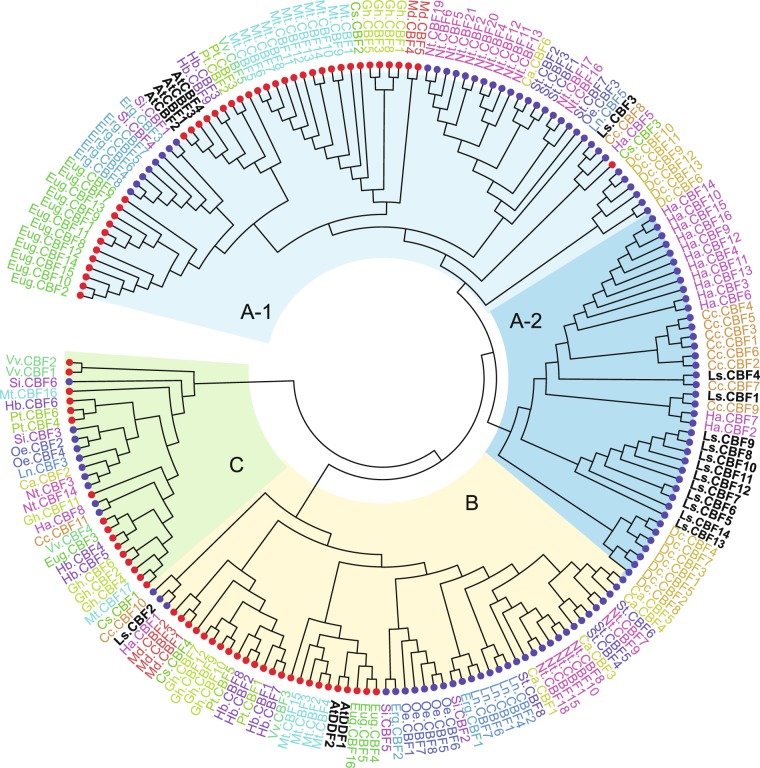
Phylogenetic analysis of 198 *CBF/DREB1* genes from 20 plant species. The phylogenetic tree was constructed using the NJ method^62^. The blue and red circles on the tree indicate Asterid and Rosid species, respectively. The *CBF* genes of Arabidopsis (At) and lettuce (Ls) are presented in black and genes from other species are color-coded according to species. The other species are: Cucumber (Cs: *Cucumis sativus*); Rubber Tree (Hb: *Hevea brasiliensis*); *Medicago* (Mt: *Medicago truncatula*); Cotton (Gh: *Gossypium hirsutum*); *Eucalyptus* (Eug: *Eucalyptus grandis*); Apple (Md: *Malus domestica*); Cottonwood (Pt: *Populus trichocarpa*); Wine grape (Vv: *Vitis vinifera*); Chili Peppers (Ca: *Capsicum annuum*); Artichoke (Cc: *Cynara cardunculus*); Carrot (Dc: *Daucus carota*); Monkey flower (Erg: *Erythranthe guttata*); Sunflower (Ha: *Helianthus annuus*); Morning glory (Ln: *Ipomoea nil*); Tobacco (Nt: *Nicotiana tabacum*); Olive (Oe: *Olea europaea*); Sesame (Si: *Sesamum indicum*); Potato (St: *Solanum tuberosum*) (See Table S1).

To determine orthologous or paralogous relationships of the *CBF/DREB1* genes from the 20 species, a phylogenetic tree was constructed using the neighbor joining (NJ) method based on the encoded protein sequences of the genes. The resulting tree categorized the genes into three clades, designated A, B, and C (Fig. 2). The three clades included genes from both Asterid and Rosid species, indicating that ancestral genes of the three clades diverged before the Asterids and Rosids separated. Clade A included the four Arabidopsis CBFs and clade B included the two Arabidopsis DDFs, while clade C did not have any Arabidopsis gene. Thus, the ancestral gene of clade C appeared to be lost in the Arabidopsis lineage. Clade A could be further divided into two subclades, A1 and A2. A1 included genes from both Asterid and Rosid species, whereas A2 included genes only from the Asterid species. Thus, subclade A1 appeared to predate a separation of the Asterids and Rosids and be ancestral to the paralogous A2 subclade (Fig. 2).

The lettuce *CBF/DREB1* genes were distributed unevenly over the clades. Clade A had 13 lettuce *CBF/DREB1* genes and clade B had only one gene, *LsCBF2*, while Clade C had no lettuce genes (Fig. 2). This result suggested that the 13 LsCBFs in clade A were orthologous to AtCBFs, and LsCBF2 was orthologous to AtDDFs, whereas, like Arabidopsis, the ancestral gene of clade C seemed to be lost in the lettuce lineage. Within clade A, most of the lettuce genes (10) formed a distinct monophylogenetic group while the two genes, *LsCBF1* and *LsCBF4*, were clustered with those of the Asteraceae family–artichoke (Cc) and sunflower (Ha)–to which lettuce also belongs. This topology was also observed among other plant species. For instance, the majority of the genes of artichoke (Cc), sunflower (Ha), and carrot (Dc) within subclade A2 were formed into separate phylogenetic groups by species, and likewise were genes of *Eucalyptus* (Eug), monkey flower (Erg), Arabidopsis (At), *Medicago* (Mt), and tobacco (Nt) in subclade A1 and genes of cotton (Gh), morning glory (Ln), and olive (Oe) in the B clade (Fig. 2). The results implied that the paralogous duplication in the *CBF/DREB1* gene family occurred in each species lineage.

### Chromosomal locations and tandem duplication of the lettuce *CBF/DREB1* genes

**Figure 3:**
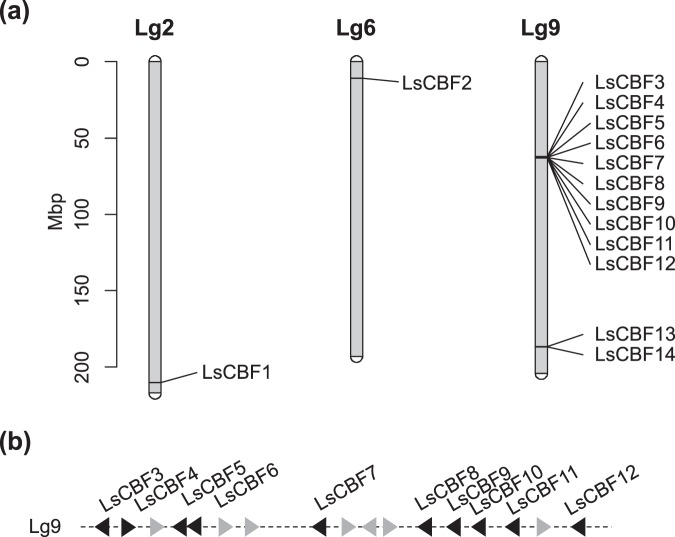
Chromosomal location of lettuce *CBF/DREB1* genes. (**a**) The genes are located over the three linkage groups: Lg2, Lg6, and Lg9. (**b**) Schematic representation of the ten genes located in a tandem cluster on Lg9. Black triangles indicate the *CBF* genes and grey triangles indicate non-*CBF* genes.

We examined the chromosomal locations of the 14 lettuce *CBF/DREB1* genes. The *LsCBFs* were unevenly distributed: chromosome 2 and 6 contained only one *LsCBF* gene, while chromosome 9 contained 12 of *LsCBFs*. Of the 12 *LsCBFs*, 10 genes were located in a cluster (Fig. 3), suggesting tandem duplication. In support of this idea is that the tandemly arrayed *LsCBF* genes (i.e., LsCBF5–12) fall into a single clade in the phylogenetic tree (Fig. 2). Thus, these results suggest that those genes on chromosome 9 evolved through tandem duplication in the lettuce lineage.

To see if selective constraints acted on the duplicated genes, we examined Ka/Ks ratio using full-length protein sequences of the genes. The pairwise comparison between the 14 *LsCBF* genes displayed a range of 0.07–0.49 of Ka/Ks ratio, and comparison of those to Arabidopsis *CBF*/*DREB1* genes displayed a range of 0.08–0.18, which are significantly lower than 1. The results indicated that the *LsCBF* genes were under purifying selection pressure with limited functional divergence (Table S4).

For those *CBF/DREB1* genes from the same species that fall into a phylogenetic clade as shown in Fig. 2, we investigated whether the genes were tandemly distributed in the genome. We examined the chromosomal location of the *CBF/DREB1* genes of carrot, *Medicago*, *Eucalyptus*, olive, and sunflower, in which chromosomal locations of these genes were available. We found that a majority of the genes in the five species were indeed tandemly arrayed (Fig. S6). These results support the idea that tandem duplication was a major process in the evolution of the *CBF/DREB1* gene family.

### Transcriptome analysis of the response to low temperature in lettuce cv. Salinas

**Figure 4:**
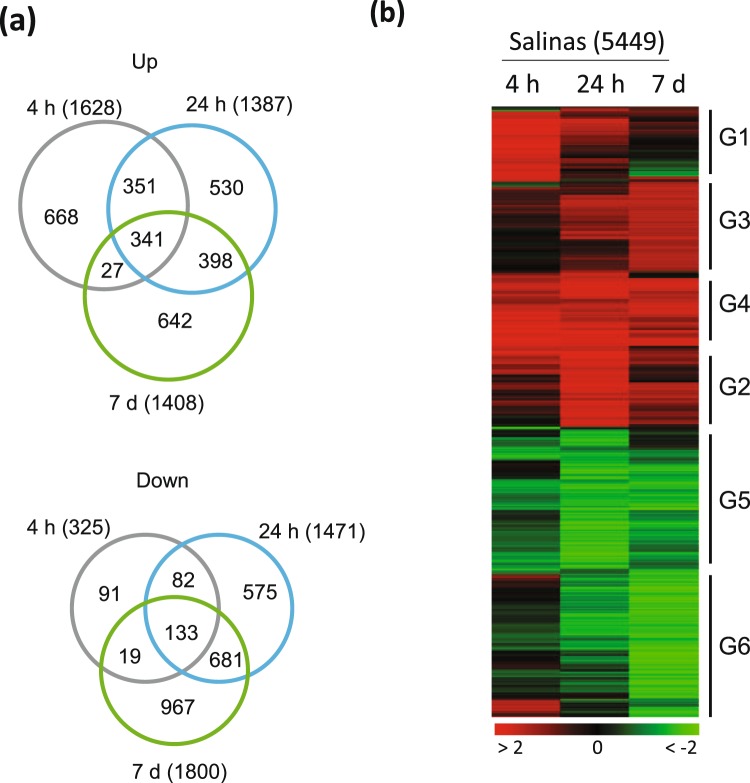
The *COR* genes of lettuce display distinctive temporal expression patterns. (**a**) Venn diagrams comparing the number of up- or down-regulated genes in plants exposed to low temperature for 4 h, 24 h, 7 d. (**b**) Heat map with differentially expressed genes in lettuce plants exposed to low temperature (4 °C) for 4 h, 24 h, or 7 days (7 d). The groups of genes labeled G1–6 show different temporal patterns of expression in response to low temperature. The color scale represents log_2_‐fold change.

To survey the global transcriptome change during cold acclimation in lettuce, we conducted RNA-seq on ‘Salinas’ lettuce plants exposed to low temperature for 0 h, 4 h, 24 h, and 7 days. We determined the *COR* genes with the criteria of fold change >2 and FDR = 0.01. We identified 5,449 *COR* genes, of which 2,957 were cold‐induced and 2,492 were cold‐repressed in lettuce plants (Fig. 4a; Table S5). The greatest number of genes (1,628) were significantly upregulated at 4 h, followed by 1,408 genes at 7 days and 1,387 genes at 24 h, whereas 1,800 of genes were significantly downregulated at 7 days, followed by 1,471 genes at 24 h, and 325 at 4 h (Fig. 4a). Venn diagram analysis indicated that 341 genes were commonly upregulated at all three time-points, which accounted for 21–25% of the upregulated genes at the three time-points, whereas only 133 genes were commonly downregulated at all three time-points, which accounted for 41% at 4 h, 9% at 24 h, and 7% at 7 days, respectively. These results also indicated that there were substantial differences in the *COR* gene sets between the different time-points. However, the differences could be due to the arbitrary cut-offs used to define the *COR* genes. To determine whether there were distinctive temporal expression patterns for the *COR* genes, we conducted a hierarchical clustering on the transcript levels of the *COR* genes. The heat map showed that there were indeed groups of genes with different temporal expression patterns. According to the time-points of peak expression, the *COR* genes could be divided into six groups: the cold-induction of G1 genes peaked at 4 h, G2 at 24 h, G3 at 7 days, and G4 throughout the cold treatment; cold-suppression of G5 and G6 genes peaked at 24 h and 7 days, respectively (Fig. 4).

Enrichment analysis of GO biological functions indicated that the G1 and G2 genes were enriched in functions relating to stresses including “defense response”, “response to stress”, “response to wounding”, and “proline biosynthesis process”. The G3 genes peaking at a later stage of cold treatment showed a significant enrichment in functions associated with protein synthesis including “rRNA processing,” “RNA processing,” and “tRNA processing” (Table 2**)**. The G4 genes that were upregulated throughout cold treatment were highly enriched in functions associated with protection from high-light intensity including “response to UV-A”, “cellular response to high light intensity,” “response to light stimulus,” and “photoprotection.” In the case of downregulated *COR* genes, the G5 genes that were suppressed most at relatively earlier stage of cold acclimation, i.e., 24 h, were enriched in functions associated with active growth including “hormone-mediated signaling pathway,” “cell wall organization or biogenesis,” and “signal transduction,” while the G6 genes that were suppressed most at later stage of cold acclimation, i.e., 7 days, were enriched in functions associated with photosynthesis or metabolism including “photosynthesis light harvesting,” “oxidation-reduction process,” “metabolic process,” and “carbohydrate metabolic process.” These results suggest that lettuce plants require the coordinated regulation of different sets of genes in the process of cold acclimation.

**Table 2:** Enrichment of GO biological functions for cold-regulated genes.

Group	GO.ID	GO.Term	Target (%)	Genome (%)	P-value
G1	GO:0010200	response to chitin	6.2	0.6	5E-28
	GO:0006952	defense response	17.8	7	7E-20
	GO:0006950	response to stress	28.6	15.1	3E-18
	GO:0009611	response to wounding	4.1	0.8	1E-11
	GO:0007165	signal transduction	16.9	8.6	3E-11
	GO:0032774	RNA biosynthetic process	16.7	8.8	2E-10
	GO:0009414	response to water deprivation	4.1	1.5	5E-06
	GO:0009409	response to cold	3.6	1.4	6E-05
G2	GO:0006561	proline biosynthetic process	0.7	12	2E-05
	GO:0006560	proline metabolic process	0.7	17	1E-04
	GO:0001101	response to acid chemical	7	968	1E-04
	GO:0009415	response to water	3.4	362	2E-04
G3	GO:0006364	rRNA processing	7.7	0.9	1E-38
	GO:0006396	RNA processing	13.4	3.8	4E-30
	GO:0034641	cellular nitrogen compound metabolic	36.4	20.9	2E-24
	GO:0010501	RNA secondary structure unwinding	2	0.2	4E-11
	GO:0008033	tRNA processing	2.3	0.6	7E-07
	GO:0042254	ribosome biogenesis	1.5	0.3	2E-06
G4	GO:0070141	response to UV-A	1	0.01	7E-09
	GO:0009411	response to UV	2.7	0.5	3E-08
	GO:0071486	cellular response to high light intensity	1	0.01	1E-07
	GO:0009628	response to abiotic stimulus	13.3	7.8	2E-06
	GO:0009416	response to light stimulus	6	2.5	2E-06
	GO:0010117	photoprotection	0.8	0.1	9E-05
	GO:0009409	response to cold	3.3	1.4	3E-04
G5	GO:0009755	hormone-mediated signaling pathway	6.9	4	7E-07
	GO:0071554	cell wall organization or biogenesis	4.3	2.2	2E-06
	GO:0007165	signal transduction	12.2	8.6	6E-06
	GO:0009626	plant-type hypersensitive response	2.9	1.4	2E-05
	GO:0009734	auxin-activated signaling pathway	2.4	1.1	4E-05
G6	GO:0009765	photosynthesis light harvesting	2	0.1	3E-24
	GO:0055114	oxidation-reduction process	11.9	5.7	2E-16
	GO:0018298	protein-chromophore linkage	2	0.3	5E-15
	GO:0009768	photosynthesis light harvesting in photosystem I	1.1	0.1	2E-14
	GO:0044710	metabolic process	25.3	17	8E-14
	GO:0046271	phenylpropanoid catabolic process	1.2	0.1	1E-11
	GO:0046274	lignin catabolic process	1.1	0.1	2E-10
	GO:0005975	carbohydrate metabolic process	7.8	4.1	2E-09
	GO:0044712	catabolic process	5.8	2.8	2E-08
	GO:0006629	lipid metabolic process	8.7	5.2	2E-07
	GO:0044711	biosynthetic process	11.5	7.8	2E-06
	GO:0005976	polysaccharide metabolic process	3.6	1.7	2E-06

### Expression analysis of the lettuce *CBF/DREB1* genes under low temperature

**Figure 5:**
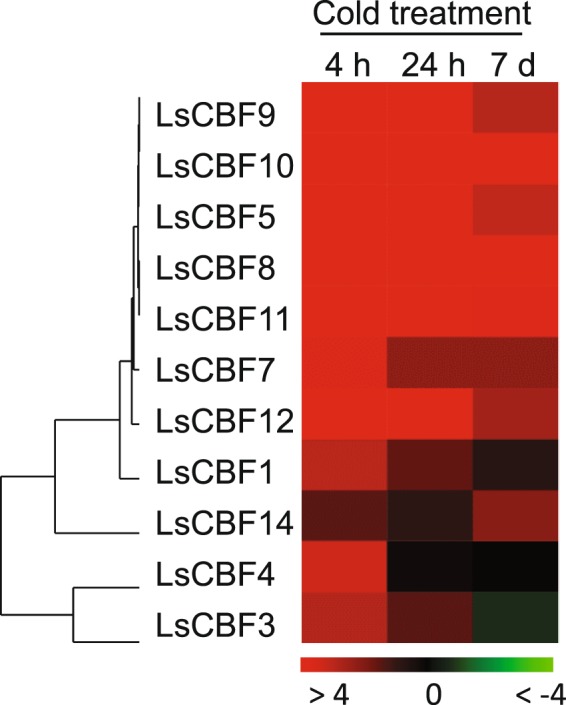
Heat map showing transcript levels of the 11 *LsCBF* genes under cold conditions. Transcript levels were measured by RNA-seq for lettuce plants exposed to 4 °C for 4 h, 24 h, or 7 days (7 d). The expression values are represented as log_2_ fold-change relative to the signals in plants grown at 20 °C.

**Figure 6:**
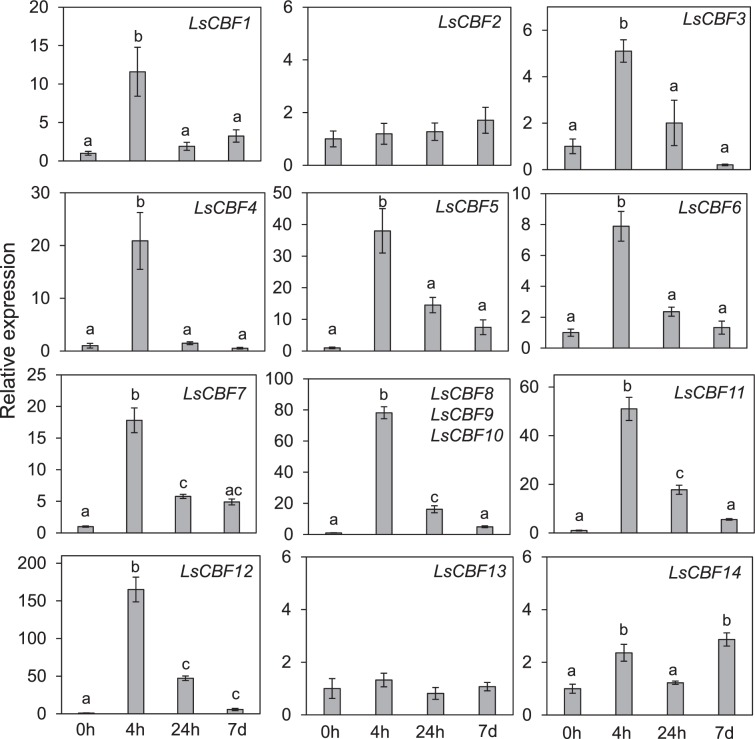
Expression of lettuce *CBF/DREB1* genes in response to low temperature. Expression levels were determined by quantitative RT-PCR in lettuce plants grown at 20 °C (0 h) or exposed to 4 °C for 4 h, 24 h, or 7 days (7 d). Average expression level for plants grown at 20 °C was set to one. *LsCBF8*, *LsCBF9*, and *LsCBF10* were amplified using a common set of primers. Error bars indicate S.E. of three biological replicates. Different letters indicate significant differences between time-points of cold treatment. Multiple comparisons of means were performed using Tukey HSD test at the 0.05 significance level^68^.

To determine their association with cold acclimation, we examined expression levels of the 14 *LsCBF* genes in plants exposed to low temperature for 0 h, 4 h, 24 h, 7 days in the RNA-sequencing. Three *LsCBF* genes–*LsCBF2*, *LsCBF6*, and *LsCBF13*–did not show any significant change in transcript levels. In contrast, the remaining 11 *LsCBF* genes exhibited a significant increase in transcript levels in response to low temperature, with their expression peaking at the time point of 4 h as do Arabidopsis *CBF1–3* genes in response to low temperature^6^ (Table S6). For instance, *LsCBF10* exhibited the highest increase with a 651-fold change (log_2_ = 9.3) relative to 0 h, followed by *LsCBF8* with 159-fold change (log_2_ = 7.3) and *LsCBF12* with 151-fold change (log_2_ = 7.2), while *LsCBF14* exhibited the least increase with a 2.8- fold change (log_2_ = 1.5) at 4 h (Fig. 5; Table S6). We confirmed the cold responsiveness of those 11 genes using quantitative real‐time PCR (qRT‐PCR) (Fig. 6). As in the RNA-seq experiments, the transcript levels of the genes peaked at 4 h of cold treatment. In addition, we tested the three *LsCBF* genes that could not be detected by RNA-seq. Consistently, the *LsCBF2* and *LsCBF13* transcript levels did not change in respond to cold. However, *LsCBF6* was significantly upregulated with its transcript levels peaking at 4 h. Thus, of the 14 *LsCBF* genes, 12 *LsCBF* genes appeared to be induced in response to low temperature, thereby suggesting a role in cold acclimation.

### Expression responses of the lettuce *CBF/DREB1* family to abiotic stress

**Figure 7:**
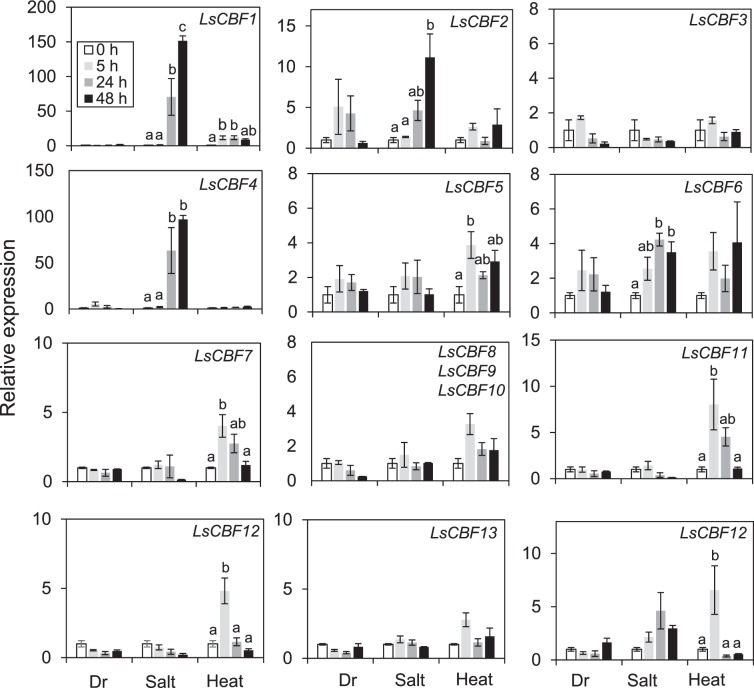
Expression of lettuce *CBF/DREB1* genes in response to abiotic stresses. Expression levels were determined by quantitative RT-PCR in lettuce plants grown at 20 °C (0 h) or exposed to drought (Dr), salt, and heat for 5 h, 24 h, or 48 h. The average expression level for plants grown at 20 °C was set to one. Error bars indicate S.E. of three biological replicates. Different letters indicate significant differences between time-points for each stress treatment. Multiple comparisons of means were performed using Tukey HSD test at the 0.05 significance level^68^.

We explored the possibility that the *LsCBFs* are associated with other abiotic stresses. We examined expression of the 14 *LsCBFs* under drought, salt, and heat stress conditions using qRT‐PCR. Of the 12 *LsCBFs* that could respond to low temperature (Fig. 6), four of them–*LsCBF3*, *LsCBF8*, *LsCBF9*, and *LsCBF10*–did not show any significant change in transcript levels under the abiotic stresses tested, whereas two–*LsCBF4*, and *LsCBF6*–were induced in response to salt stress, five–*LsCBF5*, *LsCBF7*, *LsCBF11*, *LsCBF12*, and *LsCBF14*–were induced by heat, and *LsCBF1* was induced by both salt and heat stress (Fig. 7). Thus, unlike Arabidopsis, the lettuce *CBF* genes were able to respond to multiple stresses, suggesting that they could be involved in these stresses. In addition, of the two genes that did not respond to low temperature, *LsCBF2* exhibited a significant increase of transcript levels in response to salt stress while *LsCBF13* did not show any change under the abiotic stresses tested.

## Discussion

The *CBF/DREB1* genes were identified first in Arabidopsis as key transcription factors in cold acclimation^1,42^. Since then, the CBF homologs and their contribution to enhancement of freezing tolerance have been reported in many plant species^22,28–30^. In this study, we conducted a genome-wide search for the *CBF/DREB1* orthologs in lettuce and identified a total of 14 *CBF* orthologous genes.

Our phylogenetic analysis comparing the *CBF/DREB1* genes from 19 of other plant species revealed three monophyletic groups, designated A, B, and C, and the group A can be further divided into two subgroups, A1 and A2 (Fig. 2). The A1, B, and C groups contain genes from Asterid and Rosid species, and the A2 group contains genes only from Asterid species (Fig. 2). Thus, this topology suggests that there were at least four multiplication events in evolution of the *CBF/DREB1* gene family in the Asterid lineage: three ancient events in a common ancestor of the Asterid and Rosid species and more recent one in the Asterid lineage. Many studies provide strong evidence that whole genome triplication (WGT), so called gamma event, has occurred in core eudicot lineage that is placed before the split of Asterids and Rosids^43–45^. Thus, the ancient three multiplications (i.e., A, B, and C groups) in the *CBF/DREB1* gene family might result from the WGT in core eudicot lineage. This notion is further supported by the observation that the genes from different clade groups are mostly located in different chromosomes in the diverse plant species. For example, Arabidopsis AtCBF1–3 in clade A are in chromosome 4, whereas AtDDF1–2 in clade B are in chromosome 1; lettuce genes in clade A and clade B are located in chromosome 9 and 6, respectively; and so do genes from carrot, *Medicago*, sunflower, olive, and *Eucalyptus* (Figs. 2, S6). As gene duplication could provide the raw genetic material for functional novelty^46^, one of the ancient multiplications resulted in the functional divergence of ancestral genes of CBFs and DDFs, two subgroups of the *CBF/DREB1* family that are involved in different abiotic stresses^1,33,34^: the four AtCBFs fall into group A and the two AtDDFs fall into group B. With regard to the lettuce *CBF/DREB1* genes, LsCBF2 is the only one that belongs to the DDF orthologous phylogenetic group (Group B in Fig. 2) while all other LsCBFs belong to the CBF orthologous phylogenetic group (Group A in Fig. 2). Consistent with the orthologous relationships, *LsCBF2* exhibited a responsiveness to salt stress but not to low temperature as *AtDDFs* do, and all the other *LsCBFs* except *LsCBF13* in the group A exhibited a responsiveness to low temperature. Furthermore, the cold-responsive *LsCBFs* exhibited a similar expression kinetics to that of Arabidopsis *CBF* genes, with expression peaking at around 4 h and reduced gradually at later time points in response to low temperature^42^, supporting the orthologous role of the lettuce *CBF* genes in cold acclimation **(**Fig. 5). However, there is a striking difference. Unlike Arabidopsis in which the members of the *CBF/DREB1* family can respond only to a particular abiotic stress^1,34^, eight of these 12 cold-responsive *LsCBFs* show significant induction by salt or heat stress (Fig. 7): two *LsCBFs* can respond to salt; five *LsCBFs* can respond to heat stress; and one *LsCBF* can respond to both heat and salt stress. The *LsCBFs* responsive to both cold- and heat-stress might be a result of adaption to a broad range of environmental temperature changes. For instance, the all members of *CBF* family in *Eucalyptus* are induced significantly by both cold and heat conditions, which would contribute to adaptation of the woody species to cold and dry climate in Australia where the species have originated^47^. Thus, the responsiveness of *LsCBFs* to multiple stresses (cold and heat; cold and salt; or cold, heat and salt**)** suggests that those genes may function as master regulator over abiotic stresses, such that lettuce could adapt to a wider range of climate conditions.

The 13 LsCBFs in the group A are asymmetrically distributed over the subgroups A1 and A2: subgroup A1 contains one gene, *LsCBF3*; and subgroup A2 contains the remaining 12 LsCBF genes (Fig. 2). Within the subgroup A2, the LsCBF1 and LsCBF4 are more closely related to genes from the other Asteraceae species–sunflower and artichoke–than to any other genes of lettuce, whereas the other 10 lettuce genes are more closely related to each other (Fig. 2). Moreover, eight of these *LsCBFs* are located in a tandem array in chromosome 9 (Fig. 3), which suggests that these genes evolved through tandem duplication in the lettuce lineage. This notion is further supported by the fact that those genes in tandem array are more similar to each other in protein sequence: the tandem genes display a range of 68–98% identity while they display a range of 41–59% identity in comparison with the other *LsCBFs* (Fig. S5). This tandem duplication of *CBF/DREB1* genes is also observed in other species including Arabidopsis^2^, tomato^48^, barley^22^, carrot, *Eucalyptus*, *Medicago*, olive, and sunflower (this study). Taken together, these results suggest that tandem duplication has played a significant role in the expansion of the *CBF/DREB1* gene family across diverse plant species. In addition, our phylogenetic analysis shows that the duplicates of different species are distributed over different phylogenetic clades, i.e., groups A1, A2, B, and C in Fig. 2. Duplicates of *Eucalyptus* (Eug) or *Medicago* (Mt) are in group A1 and those of sunflower (Ha), carrot (Dc) or lettuce (Ls) are in A2 while those of olive (Oe) are in group B. Thus, it seems that the tandem duplications in different species occurred independently from rather phylogenetically distinct ancestral genes. Sandve and Fjellheim^49^ provided evidence through phylogenetic and molecular clock analysis that the expansion of *CBF/DREB1* family in the Pooideae family species coincides with a global super-cooling period at the Eocene–Oligocene boundary (33.5–26 Ma), suggesting that the expansion resulted from the adaptation to the cooling climate. It is intriguing to investigate whether the diverse plant species has undergone the lineage-specific tandem duplications as a result of adaption to the supercooling climate.

Duplication is widely believed to be a major source of genetic variation through sub- or neofunctionalization that can lead to species divergence^50^. Generally, functional redundancy created by gene duplication allows the genes to accumulate mutations, leading to increase of divergence and subsequently expansion of the gene family. However duplicate genes can also be preserved by natural selection. Our Ka/Ks ratio analysis supports the latter scenario for the *CBF/DREB1* gene family in lettuce. The ratio is in the range of 0.07–0.49, which is significantly lower than 1, suggesting that purifying selection predominated among the lettuce *CBF/DREB1* genes. Purifying selection would eliminate deleterious mutations and preserve the ancestral function of duplicated genes, which could provide benefit with increased production of ancestral gene product through a gene dosage effect^51–53^. That most members of the lettuce *CBF/DREB* gene family are responsive to low temperatures and are induced with similar kinetics, peaking at 4 h of cold treatment, supports that duplicated *LsCBF* genes may be under this dosage effect. Recent studies of CRISPR-mediated CBF mutation in Arabidopsis^15–17^ also support this idea. The reports showed that the CBF mutations resulted in a gradual decrease in freezing tolerance with the triple *cbf* mutation exhibiting the least freezing tolerance and single mutations exhibiting a moderate reduction in freezing tolerance compared with wildtype control. The results indicate that the three Arabidopsis *CBF* genes act additively to increase of freezing tolerance and all three genes are required for full-capacity of freezing tolerance in Arabidopsis. Thus, it is worthy of noting that one lettuce *CBF* gene, *LsCBF7*, encodes a non-functional allele due to a premature stop codon, which could lead to reduced freezing tolerance in ‘Salinas’ plants. Given that the cold acclimation process is costly in cold but non-freezing environments, it is not surprising that the accessions adapted to warmer climate have sometimes a nonfunctional CBF^28,54^. If that is the case in lettuce, the *CBF* locus would be a good target for breeding and genetic engineering for improved freezing tolerance in lettuce. In addition to dosage effect, gene expression divergence may also play a role in the preservation of CBF duplicates. The *LsCBF2* is responsive predominantly to salt stress (Fig. 7), and this subfunctionalization would contribute to its retention by providing selective advantage in new environments^55^.

## Materials and methods

### Plant material and growth conditions

The lettuce cultivar ‘Salinas’ was grown on potting mix soil in pots in a growth chamber at 20 °C under a 16-h photoperiod with a light intensity of 350–400 μmol m^−2^ sec^−1^. The 18-day-old plants were used for stress treatments. For cold acclimation treatment^42^, plants were exposed to 4 °C for 0 h, 4 h, 24 h, or 7 days with a light intensity of 100 μmol m^−2^ sec^−1^. The other stress treatments were carried out for 0 h, 5 h, 24 h, and 48 h with a light intensity of 300 μmol m^−2^ sec^−1^. For salt and heat stress treatments, plants were treated with 250 mM NaCl^56,57^ or exposed to 34 °C^58^, respectively. For drought stress treatment, the excess water was removed from the pots and water was withheld up to 48 h. The 0 h samples were used as a control in all treatments.

### Identification of *CBF*-like genes from 20 plant species including lettuce

In this study, 20 plant species including lettuce were selected from the Asterid or Rosid clade and their protein databases were used to identify *CBF*-like genes. The protein databases of 19 species (except lettuce) were downloaded from the NCBI database and the lettuce protein database (genome v8: id37106) was obtained at https://genomevolution.org/coge **(**Table S1**)**. Where a gene had multiple isoforms in the protein databases, the longest protein was selected as a representative for the gene. The protein sequences of the Arabidopsis *CBF/DREB1* family genes (i.e., CBF1, CBF2, CBF3, CBF4, DDF1, and DDF2) were used as queries to search the protein databases using the BLASTP method with an E-value threshold of <1E-20. In addition, the presence of the AP2-domain was examined using the hmmscan function of HMMER3 v3.2.1 (http://hmmer.org)^59^ with the AP2 domain profile (Pfam accession, PF00847) as a query for the protein sequences selected from the BLASTP search. The protein sequences were excluded from further consideration if the AP2 domain was truncated, or the AP2 domain match E-value was greater than 1e-5. As a result, 652 *CBF*-like genes were identified from the 20 plant species (Table S2).

### Phylogenetic analysis

Multiple protein sequences were aligned using ClustalW2^60^ with default parameters, and the alignment was further inspected and manually adjusted with BioEdit^61^. Phylogenetic trees were generated based on the alignment using the NJ method in MEGA X version 10^62^ with the parameters of Jones-Taylor-Thornton model, uniform rates among sites, and complete deletion of gaps. The trees were visualized using FigTree version 1.4.3 (http://tree.bio.ed.ac.uk/software/figtree).

### Chromosomal mapping and Ka/Ks ratio calculation

Lettuce *CBF/DREB1* genes were mapped onto the nine lettuce chromosomal linkage groups according to their physical positions (bp). The R/LinkageMapView package^63^ was used to draw their locations onto the physical map of each chromosome. To estimate the evolution rate between the *CBF* genes, we calculated Ka/Ks ratios using a bioperl utility, ‘aa_to_dna_aln’ (https://bioperl.org) and ‘KaKs_calculator’ version 1.2^64^ with a method of model-average. The significance of Ka/Ks that deviated from neutrality (=1) was tested using the Fisher’s exact test, and the ratios with a P-value of at least 0.01 were considered as significant.

### RNA-seq analysis

Above-ground tissues were collected from ‘Salinas’ plants exposed to 4 °C for 0 h, 4 h, 24 h and 7 days. Total RNA was isolated for each biological replicate using Plant RNeasy kit (Qiagen) and submitted to Novogene Corporation (https://en.novogene.com/) for RNA-seq library preparation and sequencing. Sequencing was performed on the Illumina HiSeq platform with 150 bp paired-end reads (http://www.illumina.com). The RNA-seq reads were mapped to the *Lactuca sativa* reference genome (version 8) using STAR v2.5.2^65^. The resulting BAM files were used to count reads at the gene-level using ‘featureCounts’^66^. Differential expression analysis was implemented using the ‘edgeR’ package^67^ in R software, version 3.5.0 (https://www.r-project.org/). Because estimates of differential gene expression can be inflated by lowly expressed genes, we included only genes with at least 0.5 read per million (<0.5 CPM) in at least two samples, which resulted in 23933 genes. Genes with a two-fold change (log2 = 1) or more and an FDR = 0.01 were designated as differentially expressed. Hierarchical clustering analyses were performed using the hcluster method of ‘amap’ in R software and the resulting clusters were visualized with ‘treeview’ (http://rana.lbl.gov/EisenSoftware.htm). The RNA-seq data have been deposited in the Gene Expression Omnibus under accession number GSE134012.

### Quantitative Real-Time PCR

Total RNA was extracted from above-ground tissues of lettuce seedlings grown on soil in pots using the RNeasy Plant Mini kits (Qiagen, http://www.qiagen.com/). cDNA was synthesized with 200 ng total RNA and random primers using the Reverse Transcription System (Promega, https://www.promega.com). qRT‐PCR was performed using fast SYBR Green master mix (Life Technologies, http://www.lifetechnologies.com). Two housekeeping genes, eukaryotic translation initiation factor 2 A (*EIF2a;Ls6g95581*) and isopentenyl diphosphate isomerase 2 (*IPP2;Ls2g17540*), were used as reference genes. Lettuce homologs of those genes were identified through the BLASTN method using Arabidopsis *EIF2a* (*At5g05470*) and *IPP2* (*At3g02780*) as queries. Primers used for qRT‐PCR are shown in Table S7. Relative expression values were calculated by ddCt method using the average of two reference genes and normalized to control treatment for fold-changes. Tukey Honestly Significant Difference (HSD) test for multiple comparisons was performed using TukeyHSD function in R environment^68^.

### Gene ontology enrichment assay

Gene ontology (GO) functional annotation of lettuce genes was conducted using the Trinotate pipeline (https://trinotate.github.io/) and custom PERL scripts. The lettuce protein sequences were BLASTP-searched against UniProtKB/Swiss-Prot, which is a manually annotated, non-redundant protein sequence database. The GO terms and biological functions for lettuce genes were derived from those of the UniprotKB database if the genes had a match with an E -value threshold of <1e-20. GO term enrichment was performed on the differentially expressed genes with the 23,933 expressed gene set as a background. The significance of enrichment was tested by a hypergeometric test, which was conducted using ‘phyper’ function in R environment^68^.

## Supplementary information

Supplementary Figure S1-6.

Supplementary Table S1-7.

## Data Availability

The RNA-seq data are available in the Gene Expression Omnibus (www.ncbi.nlm.nih.gov/geo/) under accession number GSE134012. All relevant data are included in the manuscript and the Supporting Information files.
