# Supplementary material for: Genome-wide identification and expression analysis of the CBF/DREB1 gene family in lettuce
Source: Sci Rep. 2020 Mar 31;10:5733. doi: 10.1038/s41598-020-62458-1 (PMC7109083; doi:10.1038/s41598-020-62458-1)
Supplement: Supplementary file 1 — Supplementary Figure S1-6. [file 41598_2020_62458_MOESM1_ESM.docx]

**Genome-wide identification and expression analysis of the *CBF/DREB1* gene family in lettuce**

Sunchung Park^1^, Ainong Shi^2^, and Beiquan Mou^1,^*

**Supplementary information**

Supplementary Figure S1-6

**
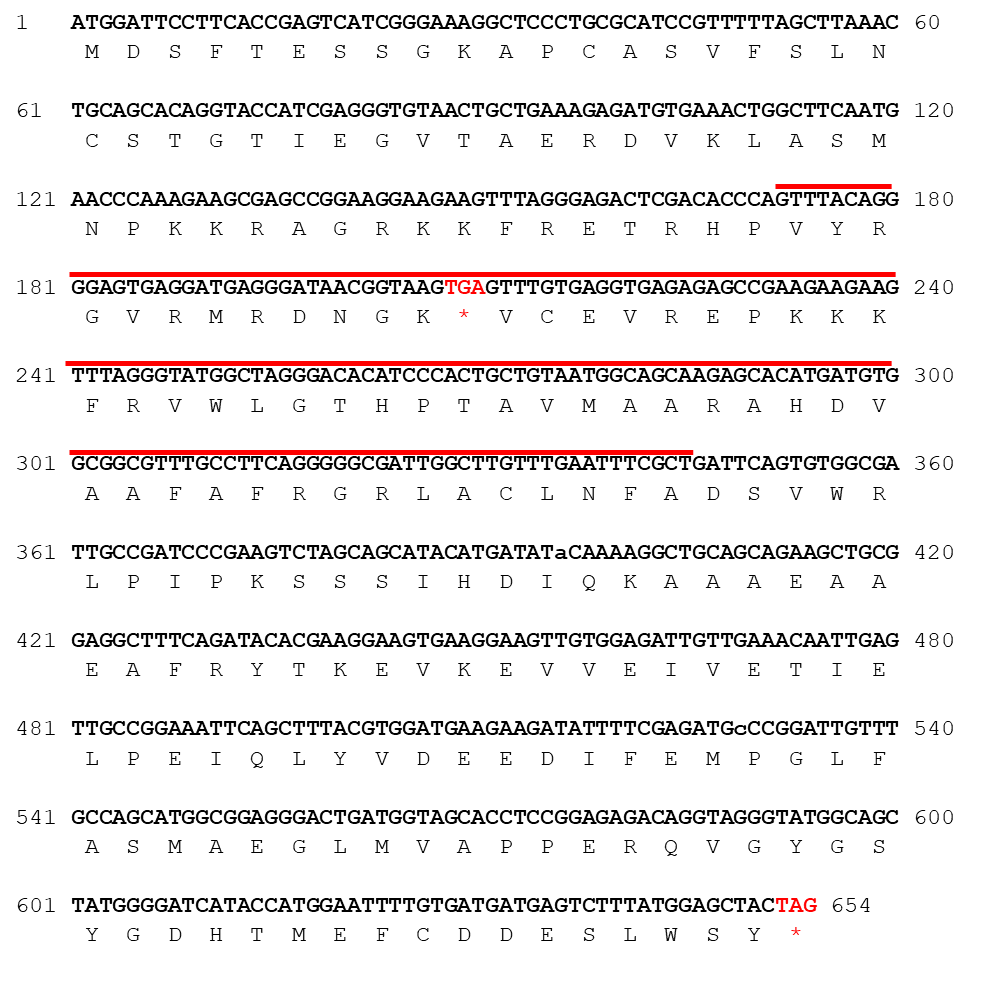
**

**Figure S1. Nucleotide and amino acid sequence of *LsCBF7* (*Ls9g54581*). Stop codons are marked in red. Red lines represent the AP2 DNA-binding domain.**

**
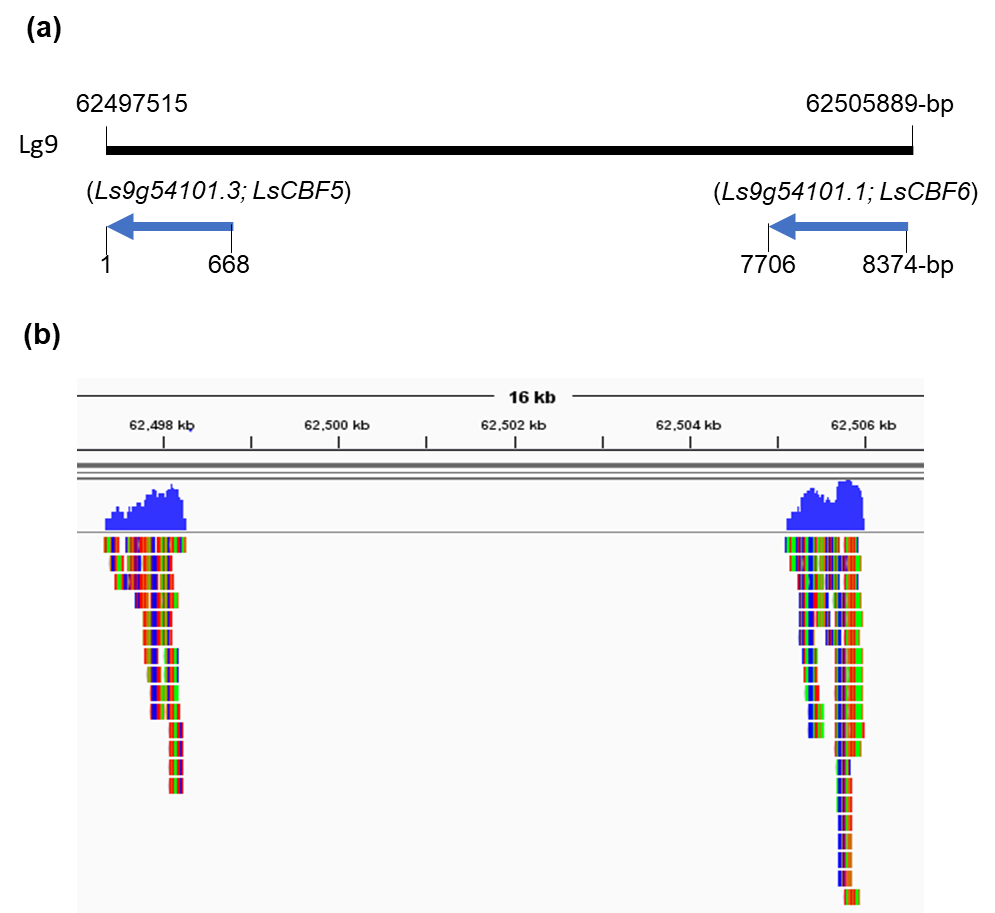
Figure S2. Schematic representation of the genomic location of *Ls9g54101* splicing variants.** (**a**) The genomic region from 62497515 to 62505889-bp in the linkage group 9 is shown. The arrows represent coding region and transcriptional direction of the two genes. Numbers at the top indicate genome coordinates; numbers at bottom, relative positions to 5’ end in bp. (**b**) RNA-seq reads from plants that were mapped to the 8-kb region encompassing the splicing variant genes. The mapped reads were visualized using Integrative Genomics Browser (<http://software.broadinstitute.org/software/igv/home>)^1^.

**
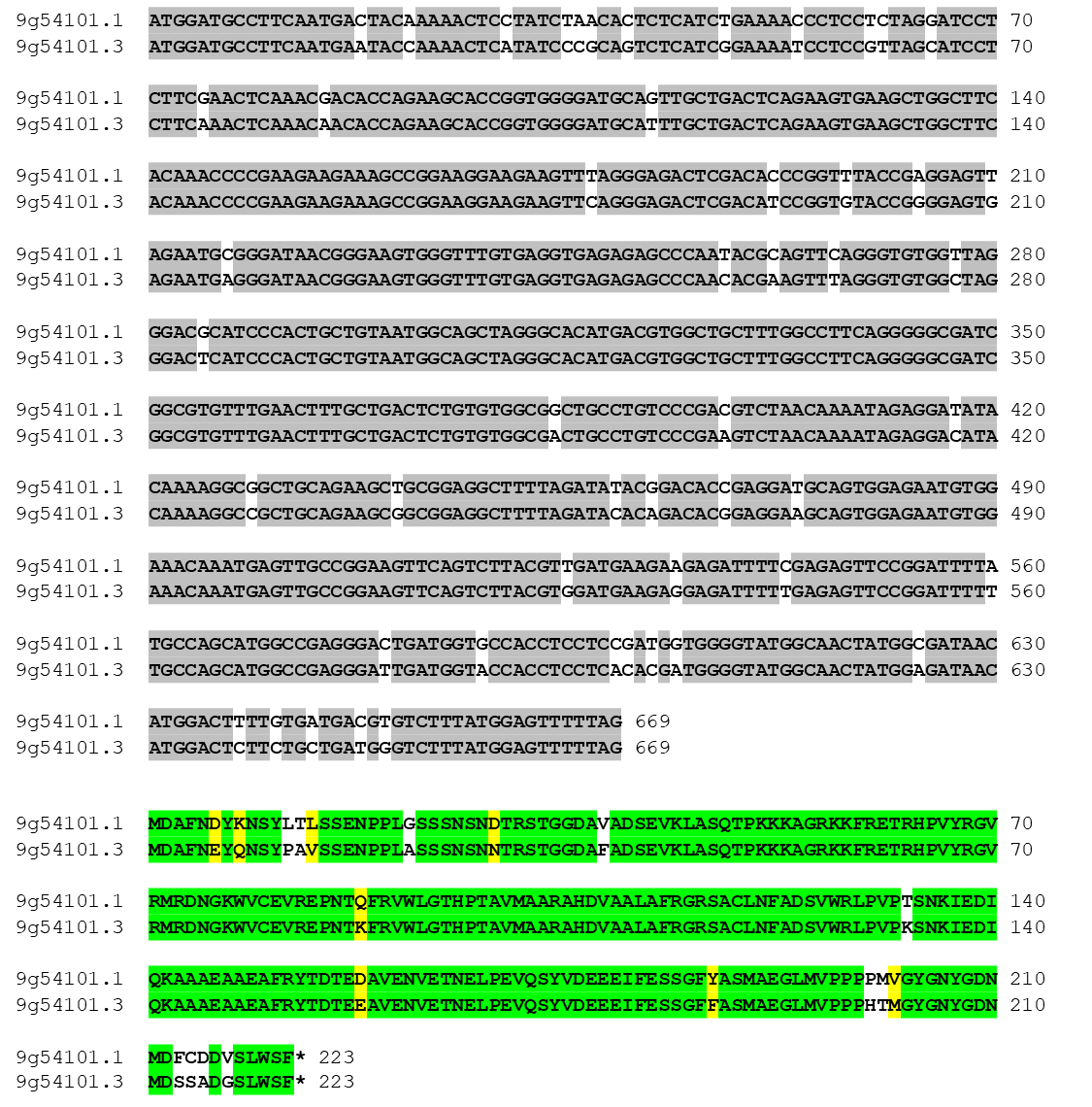
**

**Figure S3. Nucleotide and amino acid sequence alignment of Ls9g54101.1 (LsCBF6) and Ls9g54101.3 (LsCBF5).** Identical nucleotides are in grey and identical amino acids are in green, and similar amino acids are in yellow.

**
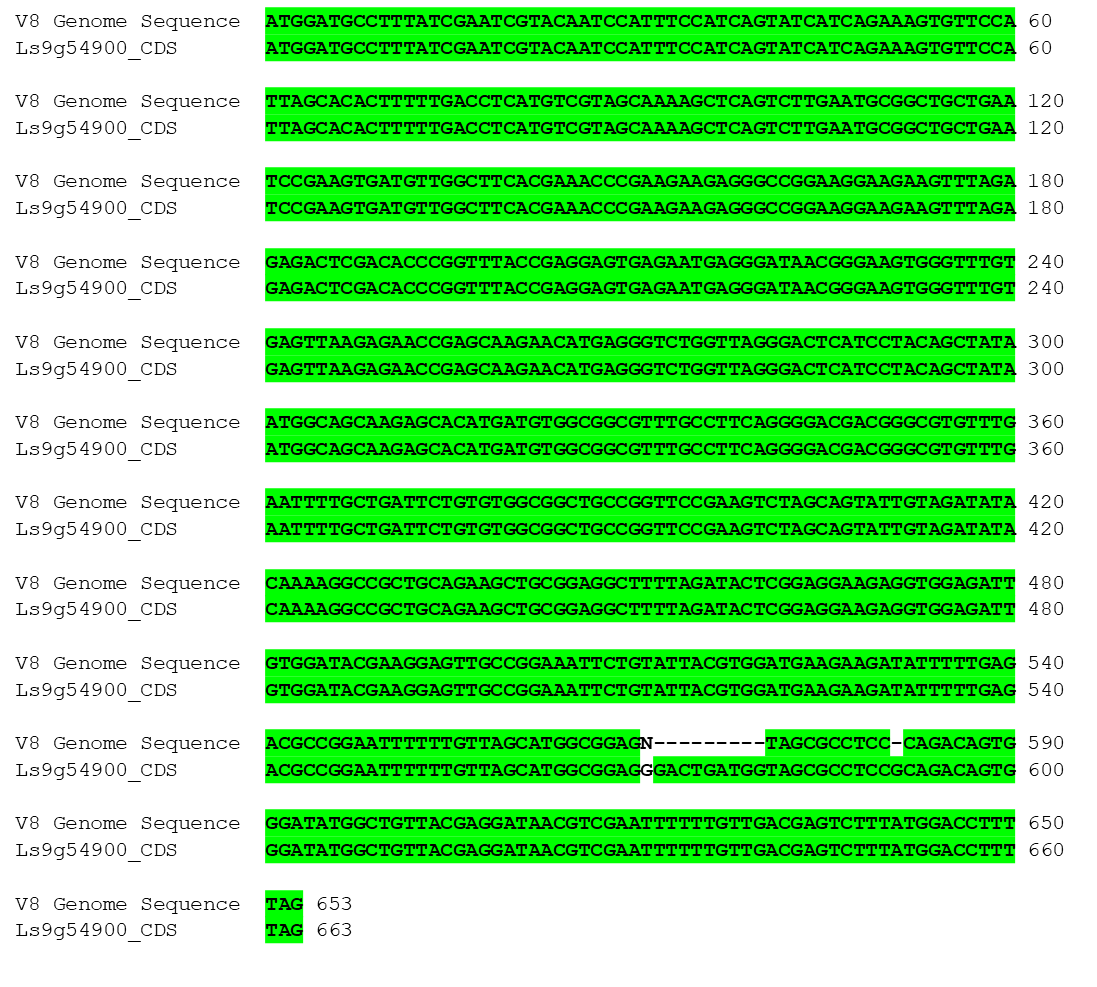
**

**Figure S4. Nucleotide sequences of *LsCBF8* (*Ls9g54900*) coding region in cv. Salinas.** The incomplete genomic sequence of LsCBF8 coding region from v8 genome was corrected by Sanger sequencing.

**
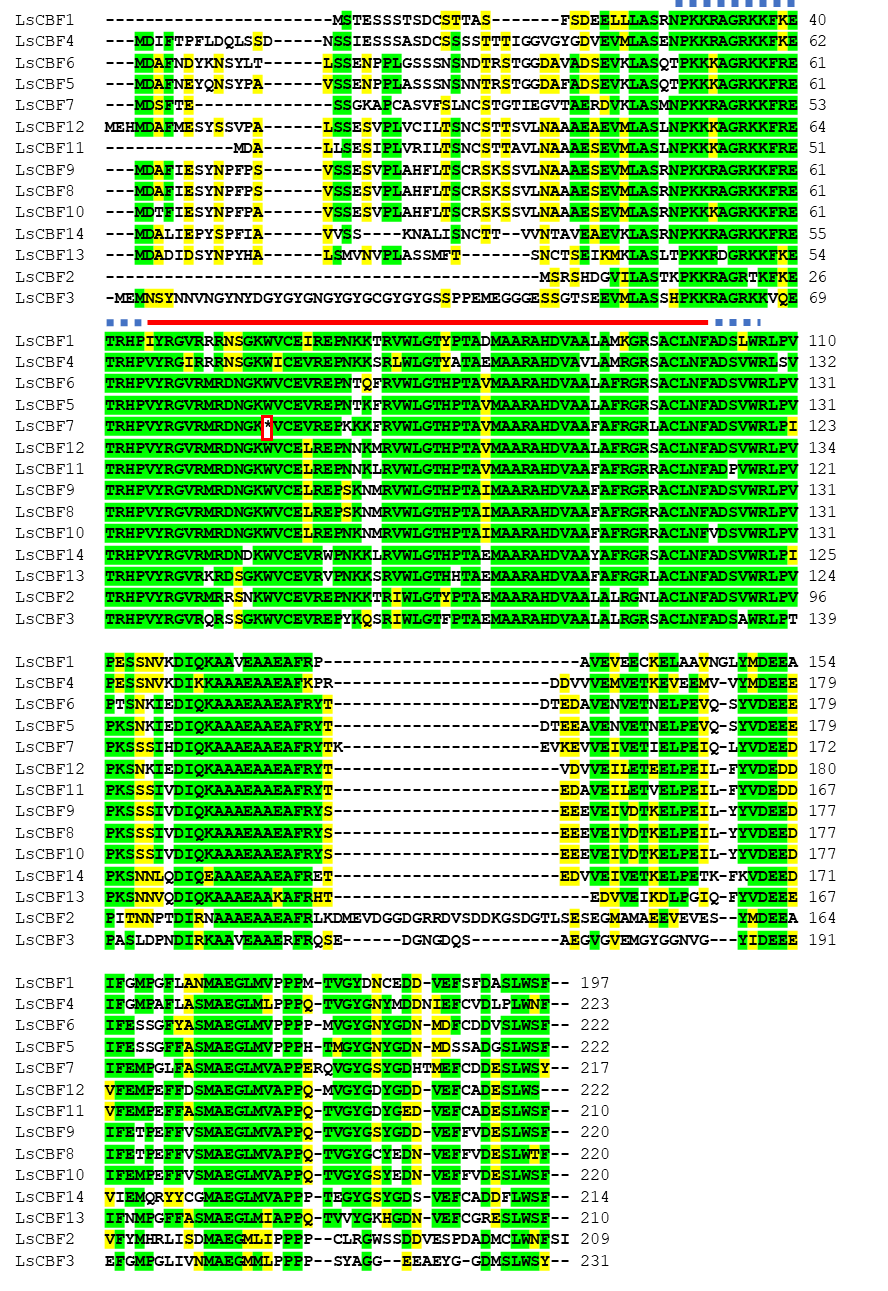
**

**Figure S5. Alignment of lettuce CBF/DREB1 proteins.** The amino acid sequences are shown for the 14 members of the lettuce CBF/DREB1 family. The AP2 DNA-binding domain is indicated by a red line and the signature sequences PKK/RPAGRxKFxETRHP and DSAWR are indicated by dashed blue lines. The red box indicates a premature stop codon in LsCBF7. Identical amino acids are in green, and similar ones are in yellow.

**
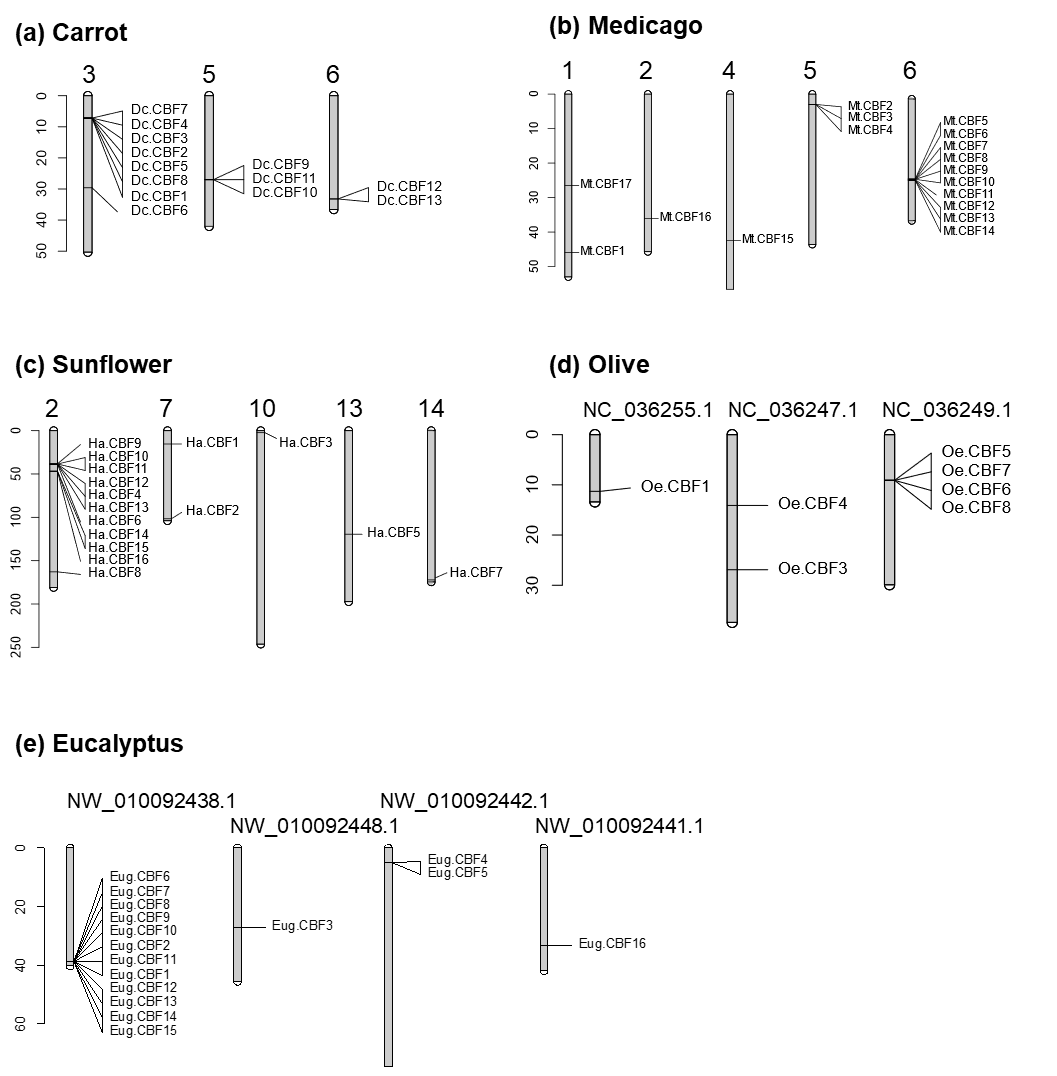
**

**Figure S6. Chromosomal location of the CBF/DREB1 family of carrot (a,** *Daucus carota***), *Medicago* (b,** *Medicago truncatula***), sunflower (c,** *Helianthus annuus***), olive (d,** *Olea europaea***), and *Eucalyptus* (e,** *Eucalyptus grandis***).** The CBF/DREB1 genes in the phylogenetic tree in Fig. 2 are mapped to chromosomes or linkage groups using the R/LinkageMapView package in R software^68^. The scales on the left side represent mega-base pairs.

**References**

1. Robinson, J. T., Thorvaldsdóttir, H., Wenger, A. M., Zehir, A. & Mesirov, J. P. Variant review with the integrative genomics viewer. *Cancer Res.* **77**, e31–e34 (2017).
